# Differential response to vibration of three forms of scoliosis during axial cyclic loading: a finite element study

**DOI:** 10.1186/s12891-019-2728-4

**Published:** 2019-08-14

**Authors:** Shaowei Jia, Ye Li, Junde Xie, Tian Tian, Shunxin Zhang, Li Han

**Affiliations:** 1 Key Laboratory for Biomechanics and Mechanobiology of Ministry of Education, School of Biological Science and Medical Engineering, Beihang University, Beijing, China; 2 School of Mechanical Engineering, Hebei University of Technology, Tianjin, China; 3 Department of Orthopedics, Peking Union Medical College Hospital, PUMC&CAMS, Beijing, China; 4 School of Medical Imaging, Tianjin Medical University, Tianjin, China

**Keywords:** Scoliosis, FE model, Axial cyclic vibrational frequency, Dynamic response

## Abstract

**Background:**

Scoliosis is a serious disease that can affect all segments of society. Few studies have investigated the response to vibration of differing sinusoidal axial cyclic loading frequencies for different forms of scoliosis in the lumbar spine.

**Methods:**

In this study, four finite element models, comprising a healthy spine, Lenke-A, Lenke-B and Lenke-C scoliosis of the lumbar S1-L1 region were developed. Modal analysis extracted resonant frequencies of the FE models with an upper body mass of 40 kg and 400 N preload. A transient dynamic analysis was performed to obtain the response to vibration of models under a sinusoidal axial loading of ± 40N at frequencies of 3, 5, 7, 9, 11 and 13 Hz using an upper body mass of 40 kg and 400 N preload.

**Results:**

The first-order resonant frequencies of healthy, Lenke-A, Lenke-B and Lenke-C spines were 9.2, 3.9, 4.6 and 5.7 Hz, respectively. A Lenke-A lumbar spine was more likely to deform at a lower vibration frequency and Lenke-C deformed more easily at a higher vibration frequency. Furthermore, the vibration amplitude in the Y-direction (left-right) was greatest and least in the Z-direction (top-bottom). The frequency of cyclic loading closest to the resonant frequency resulted in a maximum value of peak-to-peak vibrational displacement. Furthermore, the vibrational amplitudes in patients with scoliosis were larger than they were in healthy subjects. In addition, axial displacement of the vertebrae in the healthy spine changed steadily whereas fluctuations in the scoliotic vertebrae in scoliosis patients were greater than that of other vertebrae.

**Conclusions:**

Different forms of scoliosis may have different vibrational characteristics, the scoliotic vertebrae being the weak link in scoliosis under loading condition of whole body vibration. Scoliosis was more sensitive to this form of vibration. Where the frequency of axial cyclic vibrational loading of the lumbar spine was closer to its resonant frequency, the vibrational amplitude was larger. These results suggest that vibration will exacerbate the degree of scoliosis and so such patients should reduce their exposure to vibration. Clinical treatment should pay attention to the scoliotic vertebrae and reduce their vibration. These findings may assist in the clinical prevention and treatment of scoliosis.

## Introduction

Scoliosis is a three-dimensional (3D) deformation of the spine, generally developing during the period of adolescence. The principal function of the lumbar spine is to support the whole weight of the upper body, commonly approximately 40% of body weight. For loads on scoliotic spines that are asymmetric, a number of studies have reported that subjects with scoliosis exhibit a higher risk of lower back pain (LBP) than healthy individuals [1, 2]. Long-term whole-body-vibration (WBV) contributes to LBP and aggravates deformations already present in scoliosis [3]. In addition, long-term WBV has been found to increase the risk of further deformity risks for in the lumbar spine. Typical WBV exposure for train, helicopter and bus drivers has been reported to have an acceleration between 0.02 and 1.75 m/s^2^ over a range of frequencies between 2 and 25 Hz, and are directed vertically along the spine and in the anteroposterior direction [4–6]. The epidemiological literature has reported that those exposed to vibration are around 1.4 to 9.5 times more prone to back pain [7, 8]. Scoliosis patients are more likely to experience further deformities than healthy patients under a WBV loading, especially for the lumbar spine [9, 10]. Chronic axial cyclic vibration loading may lead to the spinal tissue fatigue, disc degeneration and eventually abnormal spinal deformity. Even though epidemiological studies strongly suggest that back pain can develop from whole body vibration and may be influenced by the frequency of the exposure, there has been little research to define the effects of WBV frequency for different types of scoliosis.

To understand the influence of continuous axial sinusoidal cyclic vibrational loading on the lumbar spine of healthy subjects and those with scoliosis, a considerable number of studies on the characteristics of vibration have been performed. For example, the L4-S1 model, used for comparison of the stress and strain on an axial sinusoidal loading was developed by Goel [11], revealing that cyclic loading was more dangerous than static loading. Xu [12] compared the vibrational characteristics of healthy and scoliotic spines and demonstrated that scoliotic spines suffered larger vibrational deformation than healthy spines under identical cyclic loads. Li [1] established that axial cyclic loads applied to a spine that was already deformed may induce additional rotational and scoliotic deformity and that spines with scoliosis are more sensitive to vibration than those that are healthy. Fan [13] studied the influence of variations in frequencies of sinusoidal axial cyclic loading of the lumbar spine, finding that as the cyclic frequency became close to the resonant frequency, the maximum amplitude of the vibrational displacement in the predicted dynamic response gradually increased.

Analytical studies, such as finite element (FE) methods, have been widely conducted that quantify the biomechanical characteristics of the human spine. For example, Du [14] studied the biomechanical response of lumbar facet joints with an FE model under an applied preload. Li [1] established a spine FE model and studied the dynamic response of the idiopathic scoliotic spine to axial cyclic loads. These studies proved that an FE model correctly simulated the biomechanical response of the spine and predict potential clinical treatment, indicating that FE methods provide an efficient method of evaluating geometrical and structural changes, fatigue and fracture problems of the spine, avoiding impractical or experimental treatments and computing the strains and stresses in the spinal components.

Although the influence of different axial cyclic loading frequencies and the dynamic response of the lumbar spine have been published, there are few studies of the response to different axial cyclic loading frequencies on the different types of scoliotic spine. In addition, no detailed information about the vibrational characteristics of the different forms of scoliotic lumbar spine is available and it is unclear what trends in deformation occur in scoliotic spines when subjected to WBV.

In this study, we established FE models of a healthy lumbar spine and those that exhibited three forms of scoliosis (Lenke-A, Lenke-B and Lenke-C) then subjected them to axial sinusoidal cyclic loading to predict their time-domain dynamic response. In addition, we compared their vibrational characteristics and their differences. Of the three principal effects of cyclic loading (frequency, amplitude and duration), this study focused on the frequency effect.

## Materials and methods

Three male scoliosis spines (Lenke-A, Lenke-B and Lenke-C) and a healthy spine were selected according to the scoliosis classification criteria at the imaging center of Peking Union Medical College Hospital (Beijing, China). The 3D models of the lumbar spines were developed using Mimics software based on computerized tomography (CT) images. The models were then exported to Abaqus 14.1 universal finite element software, and FE models consequently established.

**Fig. 1:**
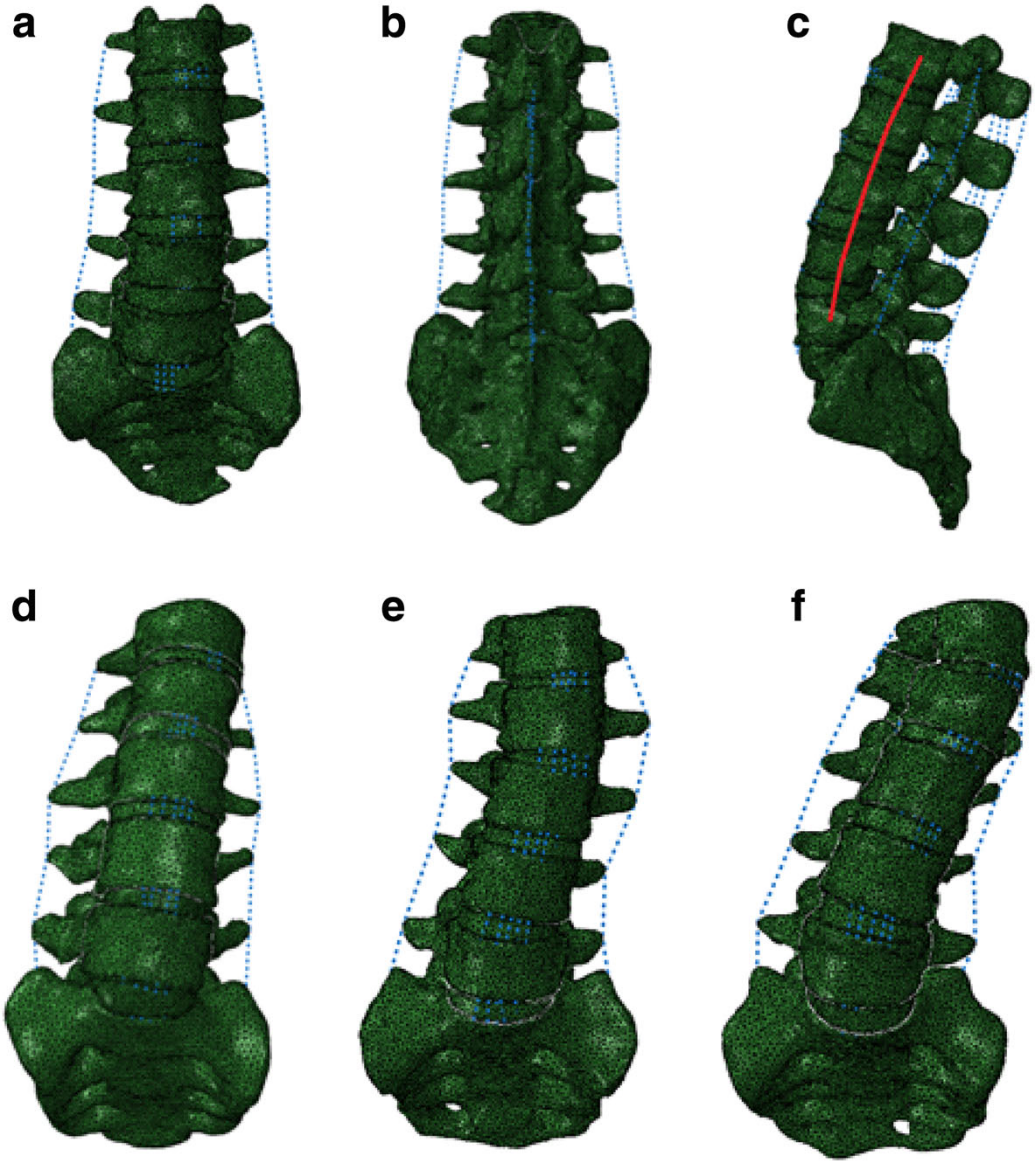
FE models of the whole lumbar spine S1-L1 combined with a compressive follower preload. **a** Front view of a healthy lumbar. **b** Back view of a healthy lumbar. **c** Lateral view of a healthy lumbar with the preload path. **d** Front view of Lenke-A. **e** Front view of Lenke-B. **f** Front view of Lenke-C

**Fig. 2:**
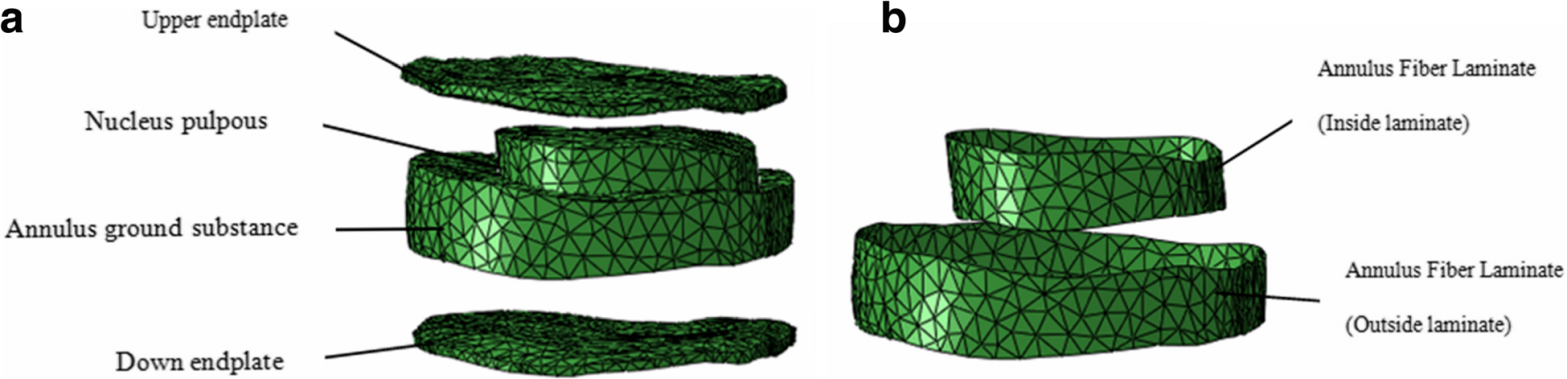
FE model of intervertebral disc. **a** Model of a complete disc. **b** Disc fibers

The FE models consisted of the vertebrae and discs of the spine. Complete FE models of the healthy and scoliotic lumbar spines are shown in Fig. 1. Vertebrae were constructed to consist of cortical and cancellous bone, and a posterior vertebral body. The cortical bone was a thin shell approximately 1 mm thick. Discs consisted of a nucleus, annulus and two endplates. The annulus fibers comprised two layers of fiber laminate, each consisting of three layers, the inner, middle and outer plies, stacked and oriented to + 30° and − 30° as shown in Fig. 2. In this study, established data from previously published literature were used for the properties of the lumbar vertebrae and discs, as shown in Table 1.

**Table 1:** Material properties of the FE models

Parts	Young’s modulus (MPa)	Poisson ratio	Reference
Vertebra			
Cortical bone	12000	0.3	[15–17]
Cancellous bone	100	0.2	[15]
Posterior body	3500	0.25	[18]
Endplate	12000	0.3	[16, 19]
Discs			
Annulus ground substance	4.2	0.45	[15–17, 20, 21]
Nucleus pulpous	1	0.499	[17, 22–24]
		E1/E2	*v*
Annulus Fiber Laminate [17, 25]			
Inside laminate	Inner ply (±30°)	360/4.2	0.3
	Middle ply (±30°)	385/4.2	0.3
	outer ply (±30°)	420/4.2	0.3
Outer laminate	inner ply (±30°)	440/4.2	0.3
	middle ply (±30°)	495/4.2	0.3
	outer ply (±30°)	5500/4.2	0.3
Ligaments		Nonlinear force-displacement curves [23, 26]	

In this study, six main ligaments were attached to the lumbar spine, namely the anterior longitudinal ligament (ALL), posterior longitudinal ligament (PLL), ligamentum flavum (LFL), intertransverse ligaments (ITL), interspinous ligaments (ISL) and supraspinous ligament (SSL). A tension axial connector was used to simulate the structure of the non-linear spinal ligaments. The parameters for each ligament have been published in previous studies [27, 28], as shown in Table 1. A follower load of 400 N [29] was applied to the FE models using an optimal path [30], simulating muscle contraction at a physiologic compressive load on the whole lumbar spine. The follower load simulated the load on the connector element [14] occurring at the center of each vertebra, with the direction of the load directed towards the center of the two adjacent vertebrae. The articulating facet surfaces were modeled using surface–surface contact elements in combination with a penalty algorithm with a normal contact stiffness of 200 N/mm and a coefficient of friction of zero. The facet cartilage layer was assumed to have a thickness of 0.2 mm. The initial gap between the cartilage layers was assumed to be 0.4 mm. The cartilage was assumed to be isotropic, linear elastic with a Young’s modulus of 35 MPa and a Poisson’s ratio of 0.4 [31].

In this study, binding constraints were established between the adjacent components in the FE models of the lumbar spine, which did not permit sliding displacement. In addition, the sacrum was entirely fixed in accordance with the anatomical characteristics of the structure of the spine, so the two sides close to the sacroiliac plane of the sacrum were set constraints and absolutely restrained all degrees of freedom of the sacrum.

The upper body mass of each subject is also an important factor for both static or dynamic analysis of the FE models. A point mass of 40 kg was used to simulate upper body weight on the top of L1. Two forms of dynamic analysis were employed in this study: modal and transient dynamic analysis. In modal analysis, a compressive preload of 400 N was applied. All degrees of freedom of the sacrum were constrained and so a lumped mass point of 40 kg was applied on top of L1 [32]. In transient dynamic analysis, on the basis of the modal analysis, an axial sinusoidal load of ± 40N was imposed on the superior surfaces of the four FE models at frequencies of 3 Hz, 5 Hz, 7 Hz, 9 Hz, 11 Hz and 13 Hz, respectively. These frequencies were chosen from the range of vibrations produced in vehicles (2–30 Hz).

## Results

### Model validation

In this study, a healthy lumbar spine was used for validation of the model. Validation of the healthy lumbar model provided confirmation of validity of the FE technique. In this way, the scoliosis models were validated. Validation of the model of the healthy lumbar spine was conducted under four different loading conditions [33]: (1) 7.5 Nm of flexion moment with 1175 N of compressive force; (2) 7.5 Nm of extension moment with 500 N of compressive force; (3) 7.8 Nm of lateral bending moment with 700 N of compressive force; (4) 5.5 Nm of axial rotation moment with 720 N of compressive force applied to level L1. The compressive force was applied as a follower load. The degrees of rotation of L1-L2, L2-L3, L3-L4 and L4-L5 are shown in Table 2. Results of this simulation were compared with those in the literature, for which the majority of the data were in agreement [34].

**Table 2:** Degrees of rotation of the model of the healthy spine under conditions of different loading

	L1-L2		L2-L3		L3-L4		L4-L5	
	Literature	This study	Literature	This study	Literature	This study	Literature	This study
Flexion	6.3	5.93	9.9	6.85	12.6	6.07	15.2	9.63
Extension	4.7	4.6	4	3.24	0.9	6.4	2	3.25
Lateral bending	4.2	4.3	5.2	3.55	5.2	6.62	3.1	3.54
Axial rotation	1	1.34	1.1	1.75	1.1	2.10	1.1	2.25

Therefore the FE model of healthy lumbar was validated, which confirmed the validity of the FE method and thus also the FE models of the scoliotic lumbar spines.

### Modal analysis

**Fig. 3:**
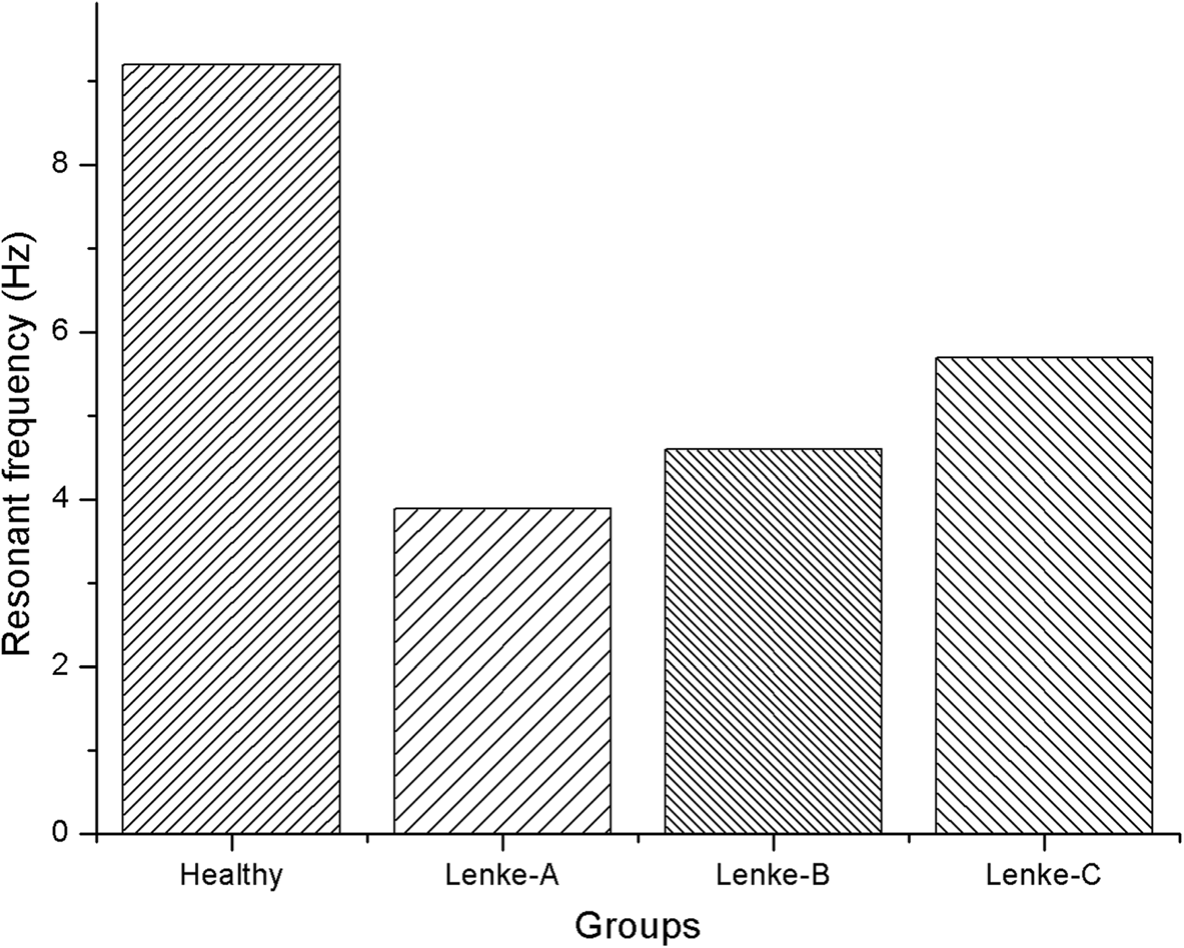
First-order resonant frequencies of the four FE model

In modal analysis, a mass point of 40 kg and 400 N preload were used to simulate upper body mass at the center of L1. First-order resonant frequencies of healthy and scoliotic lumbar models were calculated, as shown in Fig. 3, to be 9.2 Hz for the healthy model, 3.9 Hz for Lenke-A, 4.6 Hz for Lenke-B and 5.7 Hz for Lenke-C scoliosis. Thus, the fist-order resonant frequencies for Lenke-A, Lenke-B and Lenke-C were 57.6, 50 and 38% smaller than those of a healthy spine.

### Influence of cyclic loading frequency

**Fig. 4:**
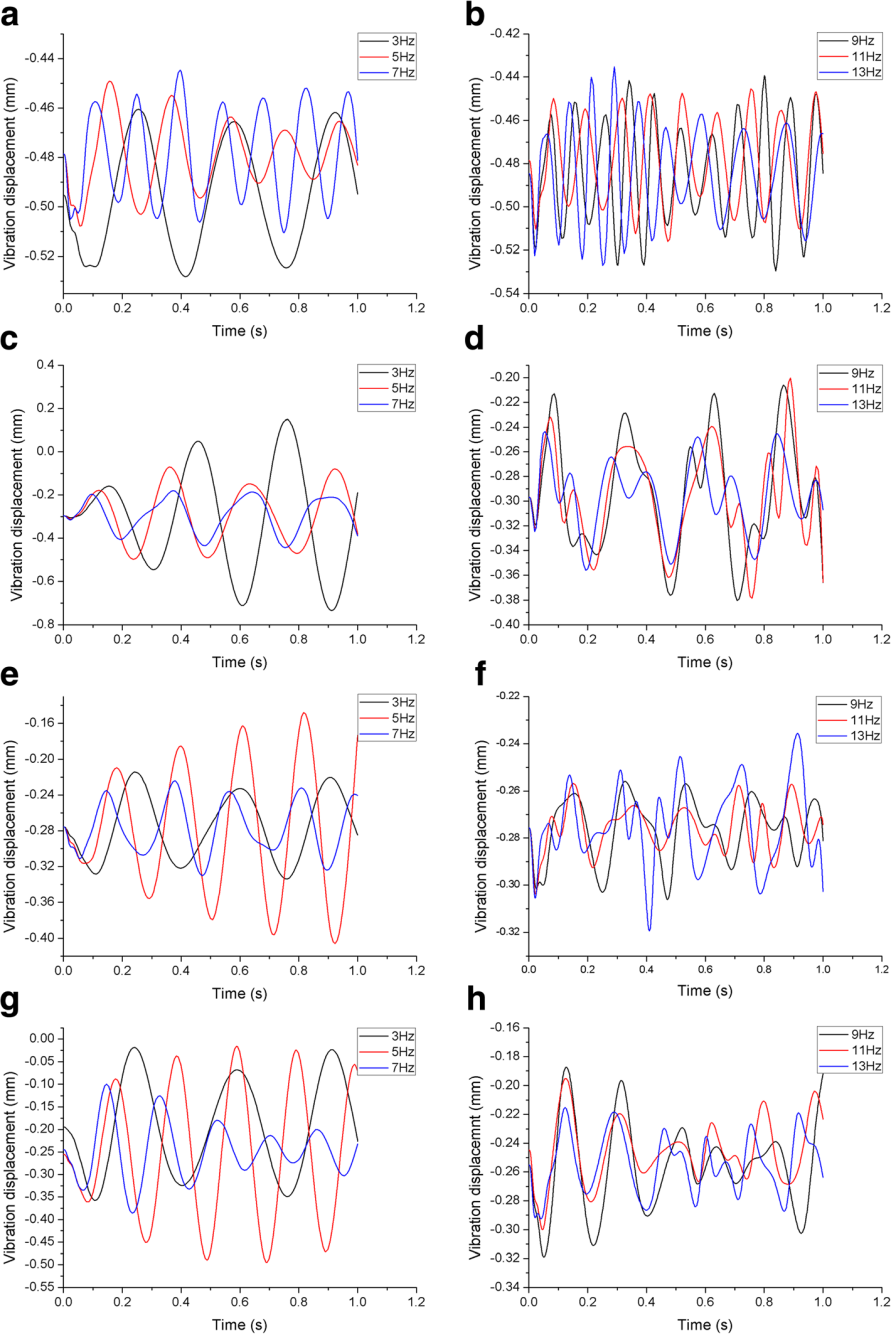
Time-amplitude curves of the four lumbar spines under sinusoidal axial cyclic loading at different frequencies. **a**), **b** Time-amplitude responses of the healthy spine. **c**, **d** Time-amplitude responses of the Lenke-A spine. **e**, **f** Time-amplitude responses of the Lenke-B spine. **g**, **h** Time-amplitude responses of the Lenke-C spine

The models were subjected to dynamic analysis under sinusoidal axial loading at various frequencies. The center of the top surface of L1 was selected as the reference point for data analysis [13]. The maximum, minimum and peak-to-peak values at the center of L1 in the axial direction of each model are shown in Table 3. The maximum vibrational displacement of the healthy lumbar was calculated at an axial cyclic loading frequency of 9 Hz. The maximum vibrational displacement of the Lenke-A scoliotic lumbar was evaluated under axial cyclic loading of 3 Hz, and Lenke-B and Lenke-C at 5 Hz. For the healthy lumbar, the amplitude of displacement when load was cycled at 9 Hz was 24.88 and 22.86% higher than at 7 Hz and 11 Hz, respectively. The amplitude of displacement for Lenke-A at 3 Hz was 53.70 and 76.80% higher than at 5 Hz and 7 Hz, respectively, for Lenke-B at 5 Hz it was 55.01 and 59.10% higher than at 3 Hz and 7 Hz, respectively and 32.01 and 44.90% higher at 5 Hz than at 3 Hz and 7 Hz, respectively, for Lenke-C. These results demonstrate that the dynamic cyclic characteristics and dynamic response were frequency–dependent. The closer the frequency of an applied load was to the resonant frequency of both healthy and scoliosis models, the greater the maximum vibrational displacement, as shown in Fig. 4.

**Table 3:** Maximum, minimum and peak-to-peak values of axial displacement at the center of L1^a^

	Healthy Lumbar			Type-A		
	7 Hz	9 Hz	11 Hz	3 Hz	5 Hz	7 Hz
Maximum	−0.4452	−0.4401	− 0.4467	0.1364	− 0.0711	− 0.2321
Minimum	− 0.5105	− 0.5269	− 0.5139	−0.7267	− 0.4707	−0.4339
Peak-to-bottom	0.0652	0.0868	0.0670	0.8631	0.3996	0.2018
	Type-B			Type-C		
	3 Hz	5 Hz	7 Hz	3 Hz	5 Hz	7 Hz
Maximum	−0.2218	−0.1523	−0.2245	−0.0234	− 0.1627	−0.1035
Minimum	−0.3318	−0.3968	− 0.3271	−0.3489	− 0.4950	−0.3673
Peak-to-bottom	0.1100	0.2445	0.1000	0.3255	0.4787	0.2638

^a^Note: peak-to-peak = maximum-minimum

**Fig. 5:**
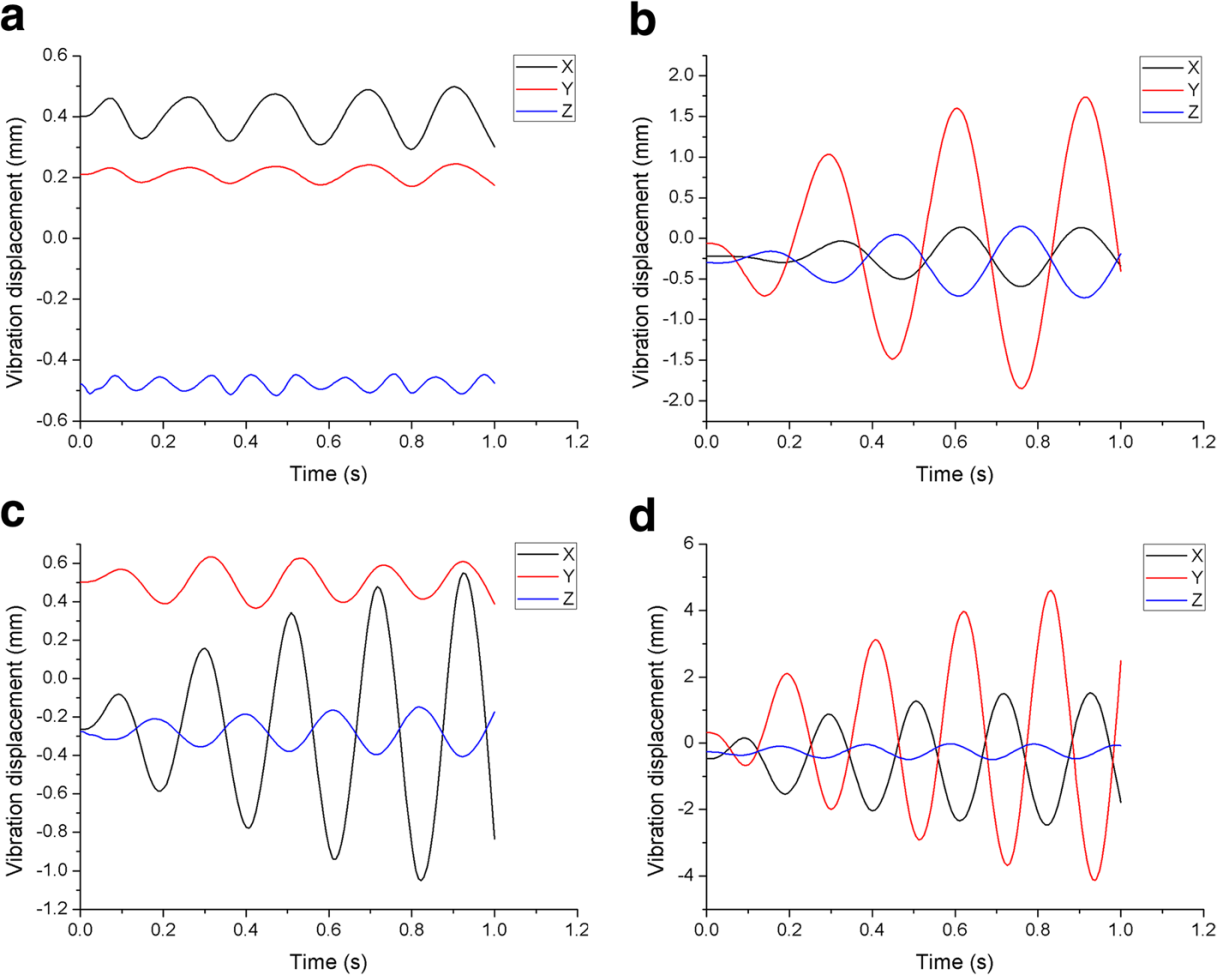
Comparisons of displacement in three directions under axial cyclic loading at resonant frequency. **a** Displacement of the healthy spine. **b** Displacement of Lenke-A spine. **c** Displacement of Lenke-B spine. **d** Displacement of Lenke-C spine

Compared with the three scoliotic lumbar spines, it was found that the vibrational displacement of the healthy lumbar was smaller in all three directions. The X-direction was defined as anterior-posterior (A-P) with anterior being positive and posterior negative. The Y-direction was defined as left-right (L-R), left being positive and right negative, and the Z-direction being vertical top-bottom (T-B) orientation, where top was positive and bottom was negative. It was observed that the maximum vibrational displacement was in the Y direction of all of the planes, with the minimum in the Z direction. This trend was similar for all four lumbar models at different frequencies. The vibrational displacement in all three directions at the frequencies closest to resonant are displayed in Fig. 5. Using the amplitude of vibrational displacement at the axial cyclic frequencies closest to their resonant frequencies, the displacement of the healthy model was only 3.68, 4.44, 15.35% of the mean of the peak-to-peak displacements of the three scoliosis models in the X, Y and Z directions, respectively.

### Vibrational characteristics of vertebrae

**Fig. 6:**
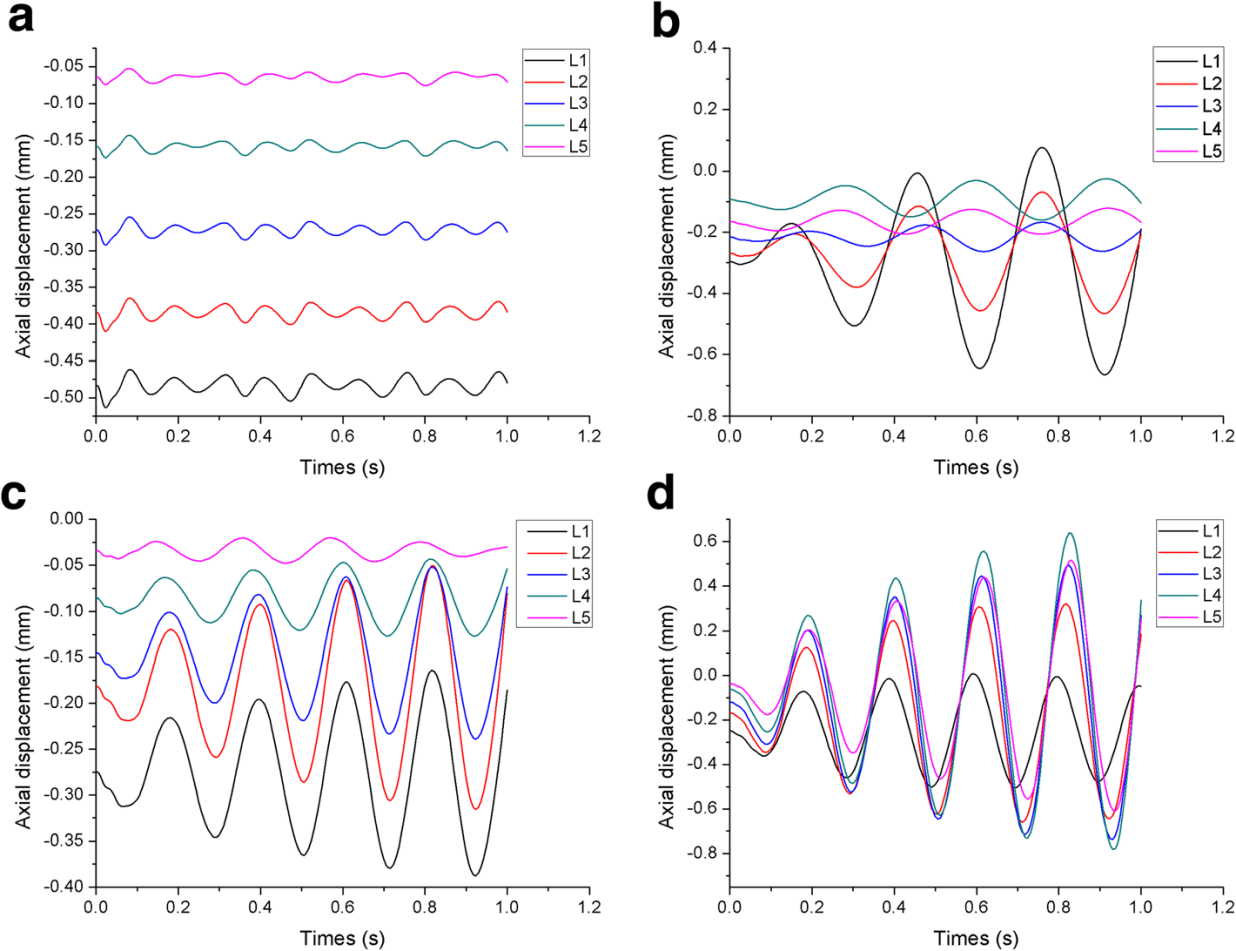
Axial displacement of the L1-L5 vertebrae of the four lumbar models. **a** Healthy spine. **b** Lenke-A scoliosis. **c** Lenke-B scoliosis. **d** Lenke-C scoliosis

The centers of the top surfaces of L1, L2, L3, L4 and L5 were selected as reference points for the data analysis. The axial displacements of the vertebrae at resonant frequencies are shown in Fig. 6. These results imply that each vertebra in the healthy lumbar spine exhibits a steady vibration with axial displacement increasing in turn from L5 to L1. The vibration curves of the three scoliosis lumbar spines exhibited severe fluctuations. The results also demonstrated that L2-L1 of the Lenke-A lumbar fluctuated greatest but L3-L5 were relatively stable, L3-L1 of the Lenke-B lumbar exhibited a large fluctuation but L4-L5 were relatively stable. However, the Lenke-C was rather different. L2-L5 exhibited a large fluctuation but L1 was relatively stable.

## Discussion

Few studies have investigated the vibrational characteristics of the different forms of scoliotic spines during WBV. The lumbar spine is an important component of the human skeleton. In addition, scoliosis is just one form of spinal disease. Many studies have evaluated the biomechanical response of the lumbar spine under various loading conditions in order to develop strategies to treat lumbar diseases, but with study of the dynamic response of the lumbar spine limited to axial cyclic loading during WBV. In this study, four FE models of human S1-L1 segments in motion were established, including one healthy and three scoliotic lumbar spines. The dynamic response of one healthy and three scoliotic lumbar spines to axial cyclic loading were compared using FE models at different vibrational frequencies.

A compressive preload of 400 N was applied so as to develop the FE models along an optimal path of follower load. This compressive preload simulated muscle contraction caused by physiologic compressive load on the whole lumbar spine. Nowadays, many studies use a follower load to simulate vibrations in vivo by the FE model or in vitro. In addition, Rohlmann [35] illustrated that a follower load can indeed represent physiological loads acting on the lumbar spine in an upright posture.

The resonant frequency of the FE models was calculated in modal analysis. The first-order resonant frequency of a healthy lumbar was found to be 9.20 Hz, a value that corresponded with the literature [12]. In addition, the first-order resonant frequencies of the three FE models of scoliotic lumbar spines corresponded with the literature [1, 12]. The resonant frequencies of lumbar spines with scoliosis were lower than in a healthy spine but with a higher vibrational amplitude, implying that scoliosis is more sensitive to vibration, confirming previous reports [1, 12, 36]. A Lenke-A lumbar is easier to deform at lower frequencies of vibration, Lenke-C at higher frequencies. Izambert [37] reported that the frequency of resonance was related to stiffness. This implies that the lumbar in Lenke-C scoliosis may be more stiff, possibly due to serious scoliosis damaging the tissue properties of the intervertebral discs leading to reduced elasticity. Xu [12] suggested that the greatest amplitude was observed in the anteroposterior direction (X-direction in this study), but the maximum value in this study was in the Y-direction. This implies that the main deformation caused by scoliosis is in the side direction and it may be sensitive to vibration. The difference may possibly be due to the models of scoliosis in this study being of a more severe degree which would lead to increased structural instability and sensitivity to vibration. Scoliosis models of varying degrees of deformity should be developed to investigate this question in the future.

On the other hand, three types of scoliotic spine were used to investigate the dynamic characteristics of scoliosis in our study, and we found that there may be a relationship between the characteristics of vibrations and type of scoliosis, including Lenke-A, Lenke-B and Lenke-C. Lenke-A and Lenke-C spines had similar deformation characteristics in three directions at their resonant frequencies. From an analysis of the vertebrae, we found that the four models had different vibrational characteristics. The healthy spine presented a very steady fluctuation due to vibrations, whereas Lenke-A exhibited larger fluctuations at L2-L1, Lenke-B at L3-L1 and Lenke-C demonstrated larger fluctuations at L5-L2. Larger fluctuations were observed in the scoliotic vertebrae. This implies that the scoliotic vertebrae were the weak link and clinicians should pay more attention to these. These results can be explained by the three scoliotic spines having different structural characteristics.

Vibrations worsen LBP, exacerbate the deformations from scoliosis and increase the risk of injury of the lumbar spine [38]. In transient dynamic analysis during axial cyclic vibrational loading, the effects of the different frequencies of vibration on the healthy lumbar spine were similar to those reported by Fan [13]. The amplitude of vibration corresponded with those studied by Goel [11] in an FE model of a healthy lumbar spine. We found that the spines with scoliosis suffered Y-direction deformation more easily and exacerbated the degree of scoliosis. This can be explained by the stability of scoliotic spines being worse than that of the healthy specimen. Similarly, the influence of the different axial cyclic vibrational frequencies on the scoliotic lumbar spines was significant, and as the frequency of the axial cyclic loading vibrations became closer to the resonant frequency, the larger was the amplitude. Fan [13] also reported a similar conclusion for axial loading vibration. Thus, frequencies of vibration close to the resonant frequency will generate lesions and exacerbate scoliotic deformation. This predicted results were found to be frequency-dependent and consistent with the notion in resonance theory texts [39] that the closer the loading frequency approaches the resonant frequency, the larger the response is. Based on resonance theory, our study calculated the amplitude of four types of lumbar spine at resonant frequencies, so clinical treatment could evolute vibration characteristics to make a appropritate treatment for scoliosis.These results suggest that vibration will worsen the degree of scoliosis and so such patients should reduce their exposure to vibration. These findings provide important evidence for the clinical prevention and treatment of scoliosis.

There are several limitations to this study. We performed only a preliminary study of the characteristics of vibration for different types of scoliosis. Only one FE model was created for each form of spinal scoliosis to study the response to vibration under axial cyclic loading. Thus, a larger number of FE models and in vitro tests should be conducted to validate the response of the spine to vibration. In future, this response should be studied in depth for increased forms of scoliosis. Moreover, the muscle tissue surrounding the lumbar spine was absent, which would affect the accuracy of the FE models. In addition, the viscoelastic properties of the discs and ligaments were neglected and the distribution of the upper body mass was not considered.

## Conclusions

In this study an important investigation of the effects of axial vibration frequency was conducted for one healthy and three types of scoliotic lumbar spines. The results indicate that the spines with scoliosis exhibited different trends of deformation. For frequencies of sinusoidal axial cyclic vibration close to the resonant frequency of each spine, the maximum value and peak-to-peak displacement of vibration of the predicted dynamic response gradually increased, this result was consist with vibration text. Furthermore, when the amplitude of the three directions of vibrations were compared, the amplitude of the Y-direction was greatest and that of the Z-direction the least. The present results suggest that the risk of lower back disorders increase when frequencies of vibration close to the spinal resonant frequency are applied and scoliotic vertebrae will undergo serious deterioration when exposed to WBV. Thus, scoliosis patients should reduce their exposure of the spine to WBV.

## Data Availability

Data is available from the corresponding author upon any reasonable request.

## Abbreviations

3D: Three dimensional
ALL: Anterior longitudinal ligament
A-P: Anterior-posterior
CT: Computerized tomography
FE: Finite element
ISL: Interspinous ligaments
ITL: Intertransverse ligaments
LBP: Low back pain
LFL: Ligamentum flavum ligament
L-R: Left-right
PLL: Posterior longitudinal ligament
SSL: Supraspinous ligament
T-B: Top-bottom
WBV: Whole body vibration
